# Methylprednisolone pulses as an initial treatment in hyperinflammatory syndrome after COVID-19 in children: evaluation of laboratory data, serial echocardiography and outcome: a case series

**DOI:** 10.1186/s40635-022-00484-1

**Published:** 2022-12-31

**Authors:** Payman Sadeghi, Mojtaba Gorji, Raheleh Assari, Fatemeh Tahghighi, Seyed Reza Raeeskarami, Vahid Ziaee

**Affiliations:** 1 Department of Pediatric Rheumatology, Pediatric Center of Excellence, Children’s Medical Center of Tehran University of Medical Science, No 63, Gharib Ave., Keshavarz Blv., Tehran, Iran; 2 Department of Pediatric Cardiology, Pediatric Center of Excellence, Children’s Medical Center of Tehran University of Medical Science, Tehran, Iran; 3 Department of Pediatric Rheumatology, Imam Khomeini Hospital Complex, Tehran University of Medical Science, Tehran, Iran

**Keywords:** COVID-19, Multisystem inflammatory syndrome in children, Kawasaki-like syndrome, Hyperinflammatory syndrome, Methylprednisolone pulse

## Abstract

**Background:**

Hyper-inflammatory syndrome in children and young adult occur 2–6 weeks after COVID-19 infection or closed contact with COVID-19 persons. In this study, the laboratory data and echocardiography and abdominal ultrasonography assessments were evaluated before and after Methylprednisolone pulse as an initial treatment of hyper-inflammatory syndrome. Therefore, the aim of this study is to assessment the clinical manifestations and laboratory data and outcome after methylprednisolone pulse as an initial treatment.

**Method:**

In this retrospective study, the demographic status, clinical features, laboratory data, echocardiography, abdominal ultrasound, treatment and outcome of 31 pediatric patients under 16 years old, with inflammatory process after COVID-19 were evaluated. The clinical assessments, laboratory data, sonography and echocardiography were evaluated before and after methylprednisolone pulse. The patients were divided in two age group < and ≥ 7 years old and the clinical manifestations were compared with each other. The Mann–Whitney *U* test was used to assess the difference in quantitative variables between two groups. To compare pre- and post- treatment values, Wilcoxol test was used. To assess the correlation between qualitative variables chi-square test was used. The level of significant was considered 0.05. These patients with fever and hyper-inflammation features admitted to the referral pediatric rheumatology ward in Children Medical Center of Tehran University of medical sciences, from April 2020 to May 2021 were assessed.

**Result:**

The mean age ± SD were (5.94 ± 3) and 51.6% (16) patients were male and 48.4% (15) patients were female. The most documented of previous COVID infection were antibody positive in about 27 (87%) patients. Moreover, 1 (3.8%) was PCR positive, 2 (7.7%) were positive in both PCR and serology and 3(11.5%) had closed contact with COVID-19 patients. About 9(29%) of patients were admitted in Intensive Care Unit (ICU). There were significant correlation between days of delay in starting treatment and ICU admission (*P*-value = 0.02). The mortality rate was negative in patients and no re-hospitalization was documented. There were significant differences (*P*-value < 0.05) between lymphocytes, platelet, Erythrocyte Sedimentation rate, C-reactive protein, Aspartate transaminase, Alanine transaminase and ferritin before and after treatment. Skin rashes and cardiac involvement totally as carditis (myocarditis, vulvulitis and pericarditis) (33.3%) and coronary involvements (53.3%) were the most prominent initial presentation in patients. There were near significant correlation (*P*-value = 0.066) between ferritin level and carditis before treatment. Cervical lymphadenopathy was seen significantly more in ≥ 7 years old (*P*-value = 0.01).

**Conclusion:**

Multisystem inflammatory system in children as a hyperinflammatory syndrome could be treated with first step methylprednisolone pulse with decreasing inflammation in laboratory data and cardiac involvements and good outcome. Furthermore, the ferritin level may be one of the predictor of severe hyper-inflammatory syndrome leading to aggressive and urgent treatment with methylprednisolone pulse.

## Introduction

Coronavirus in children and young patients may present in different features from adult patients. Although the mortality and morbidity are higher in adult, some serious complications of SARS-COV2 can also be seen in children. About two third of infected children are asymptomatic. Some children, especially with background disease, have severe COVID infection with pulmonary involvements [1]. The other involved children or closed contacts with infected person have inflammatory features with two or more organ involvements about 2–6 weeks after COVID infection [2]. The COVID PCR is usually negative with lower pulmonary involvement in this group [1]. The severe inflammatory responses as a result of cytokine releases could be consisted of gastrointestinal, hematologic, renal, central nervous system, cutaneous, nervous system and especially cardiac manifestations such as carditis, valvulitis, coronary involvements, and even the cardiogenic shock with a hyper-inflammatory process with two or more organ involvements; named Multi-system inflammatory syndrome in children (MISC) [2].

On the other hand, conjunctivitis, mucocutaneous manifestations and coronary involvements can be seen with the same features like atypical Kawasaki disease, so called Kawasaki-like syndrome [3].

Some articles separated these two post hyper-inflammatory conditions after COVID-19, but the others considered the two as one syndrome with a hyper-inflammatory process and various organ involvements [4].

Nowadays, because of limitation in studies on urgent and life-saving management of severely features of cytokines releases, the selected treatment process are based on the treatments of hyper-inflammatory process such as macrophage activation syndrome [5, 6], Kawasaki disease shock syndrome [7], secondary Hemophagocytic Lymphohistiocytosis (HLH), toxic shock syndrome; immunomodulatory agents, such as IVIG (Intravenous Immunoglobulin) [8], Glucocorticoids [9] in low to high dose as pulse therapy and Biologics [10].

In this study, methyl prednisolone pulse for one to three days was administered as the first step of treatment in hyper-inflammatory syndrome after SARS-COV2 infection [11]. Clinical manifestations, laboratory data and echocardiography (echo) and abdominal sonography, before and after treatment and the outcome of patients 1–2 weeks after discharge were evaluated. The clinical features were compared in two groups, < and ≥ 7 years old.

## Methods

In this retrospective study, the demographic status, clinical features, laboratory data, echocardiography, abdominal ultrasound, treatment and outcome of 31 pediatric patients under 16 years old, with inflammatory process after COVID-19 were evaluated. The previous positive COVID-19 were approved by PCR (Polymerase Chain Reaction), antibodies and history of closed contact in 1–2 recent months. Since, the accurate definition for inflammatory process is not demonstrated, each inflammatory condition according to CDC [2] and WHO [12] criteria of MISC and also the Kawasaki-like manifestations in pediatric patients after COVID-19 were intended. The criteria consisted of; documented fever ≥ 24 h, at least two organ involvement of cardiovascular, respiratory, renal, neurologic, hematologic, gastrointestinal, dermatologic, laboratory evidence of inflammation, any of the following: elevated CRP(C-Reactive Protein), elevated ESR(Erythrocyte Sedimentation Rate), elevated fibrinogen, elevated procalcitonin, elevated D-dimer, elevated ferritin, elevated LDH(Lactate dehydrogenase), elevated IL-6 (Interleukin6), Neutrophilia, Lymphocytopenia, hypoalbuminemia and sever condition requiring hospitalization. The Sars-cov2 infection confirmed by positive serology, positive PCR and the history of closed contact exposure within 4 -6 weeks. Hence, the pediatric patients with fever and hyper-inflammation features were admitted to the referral pediatric rheumatology ward in Children Medical Center of Tehran University of medical sciences, from April 2020 to May 2021 were evaluated. There were excluded from the PCR positive patients admitted in infectious ward of hospital. The study were reviewed and approved by Institutional Review Board of Children Medical Center (Tehran University of Medical Sciences).The patients provided written informed consent to participate in this study.

The demographic data, laboratory data, duration from clinical manifestations to hospitalization, the duration from hospitalization to first treatment, the duration of hospitalization, were documented. Echocardiography was done for all admitted patients in first hours, the second echo was done after 2–3 days after treatment and depends on patients´ condition the others echocardiography and follow up echocardiography were done 1–2 weeks after discharge. Cardiac involvements consisted: pericarditis (The inflammation of pericardia with effusion diagnosed with echocardiography), valvulitis (The inflammation of valves that could not closed completely, become leaky, named regurgitation), myocarditis (The inflammation of myocardia known with elevated troponin/BNP (Brain Natriuretic Peptide), ck-mb and/or Decreased ejection fraction and cardiogenic shock). Carditis consisted of one or more than one inflammation of pericardia, myocardia and endocardia as vulvulitis. The coronary involvements defined as ectesia (the dilation of internal luminal diameter > 2.5 z-score without aneurysm) and coronary aneurysm (The dilation of internal luminal diameter ≥ 1.5 times more than the adjuvant segment). If the patients did not have cardiac involvement, about four normal echocardiography should be documented. All echocardiographies were done by one expert pediatric cardiologist. The abdominal ultrasound was done for all patients.

The initial laboratory data in first hours of admission such as white blood cell count (WBC), polymorphonuclear (PMN), and lymphocyte absolute count, Hemoglobin, Platelet, Ferritin, Alanine transaminase (ALT), Aspartate transaminase (AST). These laboratory data were named as WBC1, PMN1, and so forth. The same laboratory data were also documented one day after methylprednisolone pulse named as WBC2, PMN2, and so forth. The dynamic changes in these variables were also calculated by subtracting the second from the first.

The other laboratory data such as fibrinogen, procalcitonin, Interleukin 6, the cardiac enzyme, Amylase, Lipase, uric acid, D dimer and the others were documented as an initial laboratory data before treatment.

According to prevalence of Kawasaki disease in < 5 years old with lower expansion to 7 years and the prevalence of MISC in teenage ages with CDC definition under 21 years old [13].

According to Children Medical Centre (CMC) protocol of Tehran University of Medical Sciences Methylprednisolone pulses 30 mg/kg/dose infused in one to three doses were prescribed as first line treatment [11]. oral prednisolone were continued 2–3 weeks after discharged and tapered gradually according to patients conditions in each visit. If the coronary arteries were involved, according to (AHA2017) [13] guidelines, IVIG 2gr/kg were initiated.

The cardiac outcome, mortality and morbidity were followed for one month.

### Statistical analysis

The statistical analysis was performed with SPSS version 20 (SPSS Inc., Chicago, IL). The Mann–Whitney *U* test was used to assess the difference in quantitative variables between two groups. To compare pre- and post- treatment values, Wilcoxol test was used. To assess the correlation between qualitative variables chi-square test was used. The level of significant was considered 0.05.

## Result

Thirty one patients were included in this study. One patient was excluded because the ultimately diagnosis in following up were systemic Juvenile Idiopathic Arthritis. The characteristic data were shown in Table 1. The most documented of previous COVID infection were antibody positive in about 27 (87%) patients. Moreover, 1(3.8%) was PCR positive, 2(7.7%) were positive in both PCR and serology and 3(11.5%) had closed contact with COVID-19 patients. About 8(25.8%) patients were referred to our hospital as a tertiary center. Duration from the first day of symptoms to diagnosis was about 8.16 ± 6.16 (1–30) days. The day of initial treatment of Methyl- prednisolone pulse was about 8.8 ± 6.5 (2–30) days from the first day of manifestations. The duration of corticosteroid treatment (methylprednisolone IV + oral prednisolone) was about 10.76 ± 4.83 (16–7) days. The duration of hospitalization was approximately 6.1 ± 2.7 (3–15) days. About 9(29%) of patients were admitted in Intensive Care Unit (ICU). There were significantly different in days of delay in starting principle treatment and ICU admission (*P*-value = 0.02). The mortality rate was negative in patients and no re-hospitalization was documented. Thus, no patient had renal involvement.

**Table 1:** The demographic characteristic of patients

Age	
Mean ± SD (range)	5.94 ± 3 (0.9–12.5)
Under 7 years old	20 (64.52%)
Above 7 years old	11 (35.48%)
Gender	
Male	16 (51.6%)
Female	15 (48.4%)

The qauntitative variable were demonstrated as means ±SD (range). The qualititative variables were shown as percentage

The mean ± standard deviation (minimum –maximum) of laboratory data were shown in Table 2. According to Table 2 there are significant differences (*P*-value < 0.05) between lymphocytes, platelet, Erythrocyte Sedimentation rate (ESR), C-reactive protein (CRP), Aspartate transaminase (AST), Alanine transaminase (ALT) and ferritin before and after treatment.

**Table 2:** The mean, standard deviation, maximum and minimum ranges of laboratory data and *P*-value of comparing of some laboratory data before and after treatment

	Mean ± SD (Maximum- Minimum): Before	Mean ± SD (Maximum- Minimum): After	*P*-value
WBC	11,200 ± 6060 (3420–23,000)	13,240 ± 5780 (4360–31,240)	0.63
Lymph	2000 ± 1240 (5450–340)	2750 ± 1750 (9540–750)	**0.005**
Hb	10.8 ± 1.27 (13–9)	10.8 ± 1.23 (12–8.5)	0.56
PLT	240,000 ± 20,000 (800,000–40,000)	400,000 ± 24,000 (900,000–63,000)	0.00
ESR	38 ± 20 (88–7)	26.8 ± 21 (97–7)	**0.014**
CRP	60 ± 45 (193–4)	20 ± 19 (90–2)	**0.001**
AST	67 ± 97 (550–18)	50 ± 73 (267–16)	**0.02**
ALT	59 ± 68 (300–11)	49 ± 30 (116–21)	**0.05**
Ferritin(micro/l)	20,000 ± 6400 (34,040–27)	2700 ± 250 (816–13)	**0.001**
Primary laboratory data			
Procalcitonin(ng/ml)			
IL6(pg/ml)	1.19 ± 2.96 (10–0.01)		
Uric acid(mg/dl)	3894 ± 638 (17,770–4.6)		
Bun(mg/dl)	5.35 ± 2.7 (10.2–2.20)		
Cr(mg/dl)	19 ± 16.8 (76–5)		
Alb(g/dl)	0.9 ± 0.7 (3.8–0.4)		
PT	3.5 ± 0.66 (4.6–2.4)		
PTT(seconds)	16.1 ± 7 (47.5–12.5)		
INR	39.5 ± 7.9 (30–22)		
D Dimer (mg/l)	1.27 ± 0.56 (3.69–1)		
Pro-BNP (ng/l)	479 ± 2180 (10,000–0.4)		
CPK	7934 ± 1523 (35,000–366)		
Ck-MB	468 ± 1079 (4240–5)		
Troponin I (ng/l)	41.4 ± 90 (375–3)		
Troponin T (ng/l)	2.36 ± 5.62 (21–0.10)		
Bilirubin	71.53 ± 66.3 (134–1.9)		
Amylase	0.45 ± 0.07 (0.5–0.4)		
Lipase	57 ± 66 (217–22)		
TG	51 ± 61 (234–11)		
Chol	217 ± 138 (549–25)		

The bold values reflected the significance difference between laboratory data before and after treatment

*WBC* white blood cells, *PMN* polymorphonuclear, *Lymph* lymphocytes, *Hgb* hemoglobin, *PLT* platelet, *ESR* erythrocyte sedimentation rate, *CRP* C-reactive protein, *ALT* alanine aminotransferase, *AST* aspartate aminotransferase, *ALP* alkaline phosphatase, *ALB* albumin, *Total Pr* total protein, *Bil T* total bilirubin, *Bil D* direct bilirubin, *LDH* lactate dehydrogenase, *BUN* blood urea nitrogen, *Cr* creatinine, *Na* natrium, *TG* triglycerides, *Chol* cholesterol, *PT* prothrombin time, *PTT* partial prothrombin time, and *INR* international normalized ratio

Skin rashes and cardiac involvement (33.3%) [Pericardial involvement, myocarditis and valvulitis] and coronary involvements (53.3%) were the most prominent initial presentation in patients (Table 3). One patient from another center was referred with myocarditis in first echocardiography, diffuse coronary ectasia in second echo and viral encephalopathy without good response to two IVIG infusions. So, three Methylprednisolone pulses were initiated in following echo the coronary involvement and carditis were resolved with residue encephalopathy. Hence, with initiation of Infliximab the patient´s consciousness was improved with nearly good responses.

**Table 3:** The percentage of clinical manifestations in hyper-inflammatory features of pediatric patients after COVID-19

	Patients(*n* = 31)
Hypertension	1 (3.3%)
Hypotension	4 (12.9%)
Respiratory	8 (25.8%)
Carditis	10 (33.3%)
Coronary involvement	16 (53.3%)
Conjunctivitis	13 (41.9%)
Skin rashes	17 (54.8%)
Oral lesions	11 (35.5%)
Cervical lymphadenopathy	4 (12.9%)
Diarrhea	12 (40%)
Liver involvement	2 (6.5%)
Acute abdomen	14 (45.2%)
CNS involvement	2 (6.5%)

*CNS* Central Nervous System

The echocardiography and abdominal ultra-sonography were shown in Table 4. Coronary involvement ectasia are the most presentation in all three tandem echocardiography. The initial echocardiography were normal in 14 patients, then the second echo 2–4 days after treatment were normal in 20 patients. Ultimately 28 patients had normal echocardiography in follow up echocardiography 1–2 weeks after discharge. One patient despite the progressive treatment included three Methylprednisolone pulses and IVIG, the coronary ectasia and aneurysm had not good response. So, Infiliximab (Tumor Necrosis Factorα inhibitor) was prescribed with nearly good responses in following echocardiography. About one patient had residual coronary involvement 1–2 month after discharge.

**Table 4:** The echocardiography and abdominal sonography findings of pediatric patients with hyper-inflammatory features after COVID-19

	Patients (*N* = 31)
Echocardiography 1	
Coronary ectasia	6 (19.4%)
Coronary aneurysm	2 (6.4%)
Valvulitis	2 (6.5%)
Myocarditis	1 (3.2%)
Pericarditis	2 (6.5%)
*C. ectasia* + Valvulitis	3 (9.7%)
*C. ectasia* + Valvulitis + Pericarditis	1 (3.2%)
Normal	14 (45.2%)
Echocardiography 2	
Coronary ectasia	4 (12.9%)
Coronary aneurysm	2 (6.4%)
Valvulitis	1 (3.2%)
Myocarditis	0
Pericarditis	0
*C. ectasia* + Valvulitis	1 (3.2%)
*C. ectasia* + Myocarditis	1 (3.2%)
*C. ectasia* + Valvulitis + Pericarditis	2 (6.4%)
Normal	20 (64.5%)
Echocardiography 3	
Coronary ectasia	2 (6.4%)
Coronary aneurysm	1 (3.2%)
Valvulitis	0
Myocarditis	0
Pericarditis	0
Normal	28 (90.3%)
Abdominal sonography	
Serositis	6 (19.4%)
Mesenteric adenitis	1 (3.2%)
Terminal ileitis	3 (9.7%)
Hepatomegaly	1 (3.2%)
Hydrops of gallbladder + Serositis	2 (6.5%)
Terminal ileitis + Serositis	1 (3.2%)
Mesenteric adenitis + Serositis	1 (3.2%)
Serositis + Mesenteric adenitis + Terminal ileitis	2 (3.2%)
Normal	14 (45.2%)

Echocardiography 1: were done at first day of admission. Echocardiography 2: were done 2–3 days after treatment. Echocardiography 3: follow up echocardiography were done 1–2 weeks after discharge

*CNS* Central Nervous System

There were near significant corellation (*P*-value = 0.066) between higher ferritin level and carditis. However, the significant difference were not found between CRP, ESR and coronary involvements and carditis respectively (*P*-value: 0.7, 0.38, 0.92, 0. 35). Also, there were no significant difference between Ferritin level and coronary involvement. (*P*-value = 0.74).The patients were divided in two subgroup less than 7 years old and more than 7 years old. Although the initial presentations especially the gastrointestinal manifestations in less than7 group were more than other group, there were no significant difference between two groups. Cervical lymphadenopathy in more than 7 years old were more than the other group with significant difference (*P*-value = 0.01). Thus, there were significantly different between AST level and cervical lymphadenopathy (*P*-value = 0.02). However, there were no significantly different between cervical lymphadenopathy and ALT, CRP, ESR, Ferritin, respectively (*P*-value: 0.16, 0.88, 0.2,0.47).

All the patients received methylprednisolone pulse and Aspirin and approximately 8 patients (25.8%) received IVIG. Also, Enoxaparin was prescribed for 8 (25.8%) of patients. Infliximab was prescribed in two patients because of aneurysm in coronary artery and CNS involvement.

## Discussion

The hyper- inflammatory syndrome because of severe cytokine release occur about 3–6 weeks after SARS-COV2 infection or closed contact with COVID-19. The antibodies against SARS-COV2 were more important than PCR in this study like the other articles. These antibodies probably display the function of acquired immune system against auto-antigens and lead to activation of the other immune cells; such as T-cell, macrophages, neutrophils and ultimately result in cytokine storm with multiple organs involvement [14]. With considering hyperinflammatory syndrome as a cytokine storm could be treat with methylprednisolone pulse, we evaluated the laboratory data, echocardiography results before and after methylprednisolone pulse treatment in MISC patients.

The mean age was lower than the other studies and the male to female ratio was nearly similar to other studies. The differences were due to selection of patients with one to two positive laboratory data and at least two organs involvement and echocardiography- and sonography-positive data [15, 16].

Multisystem inflammatory syndrome in children (MISC) and Kawasaki-like syndrome both illustrate the hyper- inflammatory syndrome. Some articles emphasize that MISC is a novel disease after COVID -19 pandemic in children with older age and more gastrointestinal manifestations and myocarditis [17]. On the other hand, there are some reports about more Kawasaki-like syndrome, with features of atypical Kawasaki disease and coronary abnormalities, after SARS-COV2 [18, 19].

Furthermore, there are some similarities between MISC and Kawasaki disease. Cytokine storm lead to hyperinflammatory condition with myocarditis in both of them [20]. In addition, high level of ferritin can be seen in both MISC and MAS in Kawasaki disease [20, 21]. Moreover, autoantibodies in MISC patients have some special target auto-antigens on endothelial cells and myocardial cells leading to small and medium-sized vasculitis similar to KD [22]. These auto-antigens were also reported in KD [23]. However, the exact pathogenesis of MISC still remains unclear.

The mucocutaneous involvement, conjunctivitis, lymphadenopathy are seen in both MISC and atypical KD. The cardiac manifestations such as coronary and myocardial involvement have the same presentation in MISC and atypical-KD, especially myocarditis in Kawasaki disease shock syndrome (KDSS). The KDSS in acute phase of Kawasaki disease is IVIG-resistant with higher level of inflammatory cytokines such as IL6, TNFα, and IL1 and good response to methylprednisolone pulses [7, 24]. Nowadays, the researchers work on methyl prednisolone pulses instead of IVIG treatment in acute phase of Kawasaki disease. In acute phase of Kawasaki disease, the innate immune system are involved with cytokines released as a hyper-inflammatory process [25, 26].

The mucocutaneous involvement and cardiac manifestations were the most clinical presentations. The patients were divided in two groups of less or more than 7 years old. The majority of Kawasaki disease are in the age of 2–5 years old with mucocutaneous- lymph node syndrome. The MISC patients are older with more prominent organ involvement; gastrointestinal, myocarditis and shock. The purpose was to separate these two phenomena according to special presentations. However, there were no significantly difference in gastrointestinal and cardiac involvement between two age groups. So, MISC and KD- like disease may be the similar hyper-inflammatory syndromes with wide spectrum of organ involvements [4, 27, 28]. These findings may be against assumption of difference between MISC and Kawasaki-like disease based on age ranges and organ involvements [1, 3].

Cervical lymphadenopathy was more frequently seen in ≥ 7 years old group. First-Node Kawasaki Disease (FNKD) in an unusual presentation of atypical Kawasaki disease in older ages, similar to this study [29, 30]. The FNKD patients have more hyper-inflammatory process with more coronary involvements and IVIG- resistance. In NFKD, the IL6, AST, CRP, ESR are in higher levels in comparison with other atypical Kawasaki disease [29]. This study, with evaluation of hyper-inflammatory syndrome after SARS-COV-2, found significant correlation between AST and cervical lymphadenopathy (*p*-value = 0.02). So, the reticuloendothelial system involvement may be more prominent in these age group.

The initial treatment was methyl-prednisolone pulse, in contrast with the other articles using IVIG in FNKD. There was no significant correlation between coronary involvements and cervical lymphadenopathy (*P*-value = 0.6). Therefore, the cervical lymphadenopathy may predict the IVIG-resistant hyper-inflammatory syndrome with good responses to methylprednisolone pulses. In addition, the earlier use of pulse in our study might decrease the coronary involvement [29, 30].

Macrophage activation syndrome (MAS) is a cytokine storm with increased level of ferritin produced by activated macrophages [5, 31]. Secondary HLH like MAS, primary HLH and probably MISC, may initially have different mechanisms in stimulation of inflammatory pathway, nevertheless all of them lead to activated macrophages, neutrophils, histiocytes and the other cells and subsequently elevated ferritin level and ultimately cytokine storm with some organ damages [32]. In addition, the acute phase of Kawasaki disease is presented with activation of innate immune system as a cytokine storm, especially in KDSS with myocarditis and elevated ferritin level [7, 33].

The correlation between ferritin level and carditis were near-significant. With more sample size, the correlation can be significant. So, Ferritin level may be a valuable predictor of carditis, with effect on decision for aggressive treatment.

One to three doses of Methylprednisolone pulses were the initial treatment. In previous study, methylprednisolone pulse was not prescribed alone without IVIG [10, 17, 34–37]. In some studies, Biologic treatments were used in resistant patients more than this study [10, 17, 34]. IVIG and low dose glucocorticoid were the principle treatment in these studies [35–37]. However, one study demonstrated no significant differences between IVIG and glucocorticoid and high dose glucocorticoid alone as first-line treatment [39]. In our study, the biologic treatment were prescribed in only two patients and the mortality were zero. However, one patient presented persistent aneurysm in 1–2 weeks following echocardiography.

There were significant differences between inflammatory laboratory data before and after methylprednisolone pulses treatment with rapid decrease within two to three days after methyl prednisolone pulses. On the other hand, the delayed initiation methyl prednisolone pulses were associated by more ICU admission. So, decreasing inflammatory parameter might have an effect on low rate of complications and lower use of Biologic treatments [36–38].

The mainweakness of this study were low sample size and retrospective design without control group. Higher sample size and prospective study would be more valuable. Furthermore, the presence of control group will yield more accurate result. Further clinical trials for evaluation of selected treatments should be considered.

In conclusion,.we have shown methylprednisolone pulse as the first step of treatment in MISC patients with considering the hyperinflammatory syndrome could decrease the clinical, laboratory and cardiac inflammation with good outcome. However, control group with more sample size would be more valuable in future studies.

Furthermore, the ferritin level may be one of the predictor of severe hyper-inflammatory syndrome leading to aggressive and urgent treatment with methylprednisolone pulse.

## Data Availability

The data sets study are available from the corresponding author on reasonable written request.
